# Sex-related illness perception and self-management of a Thai type 2 diabetes population: a cross-sectional descriptive design

**DOI:** 10.1186/s12902-017-0229-8

**Published:** 2018-01-30

**Authors:** Wimonrut Boonsatean, Anna Carlsson, Irena Dychawy Rosner, Margareta Östman

**Affiliations:** 1 Faculty of Nursing Science, Rangsit University, Pathum Thani, 12000 Thailand; 2 Faculty of Health and Society, Malmö University, SE 205 06 Malmö, Sweden

**Keywords:** Type 2 diabetes, Illness perception, Self-management, Sex differences, Thailand

## Abstract

**Background:**

Increased knowledge concerning the differences in the illness perception and self-management among sexes is needed for planning proper support programs for patients with diabetes. The aim of this study was to investigate the illness perception and self-management among Thai women and Thai men with type 2 diabetes and to investigate the psychometric properties of the translated instruments used.

**Methods:**

In a suburban province of Thailand, 220 women and men with type 2 diabetes participated in a cross-sectional descriptive study. The participants were selected using a multistage sampling method. Data were collected through structured interviews and were analyzed using group comparisons, and psychometric properties were tested.

**Results:**

Women and men with type 2 diabetes demonstrated very similar experiences regarding their illness perception and no differences in self-management. Women perceived more negative consequences of the disease and more fluctuation in the symptoms than men, whereas men felt more confident about the treatment effectiveness than women. Furthermore, the translated instruments used in this study showed acceptable validity and reliability.

**Conclusions:**

The Thai sociocultural context may influence people’s perceptions and affect the self-care activities of Thai individuals, both women and men, with type 2 diabetes, causing differences from those found in the Western environment. Intervention programs that aim to improve the effectiveness of the self-management of Thai people with diabetes might consider a holistic and sex-related approach as well as incorporating Buddhist beliefs.

## Background

Type 2 diabetes (T2D) has shown an increasing global prevalence in the latest decade [1, 2]. The worldwide prevalence was approximately 2.8% in 2000 [3] and increased to 9% in adults (20–79 years old) in 2014 [4]. The prevalence of T2D in Thailand has also increased annually to approximately 6.4% in 2013 [1], and is one of the five common chronic diseases in Thailand [5].

Various international studies have explored the biological risks in developing T2D between women and men [6, 7]. Additionally, there are also studies investigating the differences among sexes in psychological aspects such as distress, anxiety, and depression [8–10]. However, only few studies have investigated sex differences regarding the health perceptions and attitudes [11–14] or self-management [15] in a population with T2D.

The term “illness perception” is used both to describe a person’s cognitive and emotional response pattern and coping styles when living with the disease as well as the experience and understanding of his or her situation [16]. Additionally, the perception of being discriminated against by society due to the disease is also included in the concept of illness perception [17]. As found in contemporary research, negative emotional responses can lead people with T2D to feel overwhelmed and to find it difficult to manage their life with diabetes [18].

Self-management is often described as the way one is managing his or her life with the disease, a process of taking control of the disease through individual cognitive decision making by obtaining support from one’s family and from healthcare professionals [19]. Western studies have shown that people that have been able to take control of their diabetes have adopted efficient and comprehensive ways of dealing with the disease [20], although dietary control was found to be difficult [15, 21]. Furthermore, demographic characteristics have been seen to influence individual management strategies [22]. While women with T2D are seen to more often strictly attend to medical recommendations and take advantage of social resources, men more often rely on themselves and search for new strategies to manage their disease [15].

Research has shown that only 28% of Thai people with diabetes can manage their disease well [23]. In order to increase the number of people that are able to effectively manage their T2D, more knowledge on the part of healthcare professionals concerning the influences of illness perception and self-management is needed. Studies conducted with Westerners with diabetes have found that some differences between women and men exist with regard to their perceptions and attitudes towards T2D [11, 12, 18] and the ability to handle the disease [15]. Because there is limited knowledge in Thailand concerning the differences in illness perception and self-management among women and men, a study comparing the sexes in these aspects would be appropriate.

The aim of this study was to investigate the illness perception and self-management among Thai women and men with type 2 diabetes. An additional aim was to investigate the psychometric properties of the translated instruments used.

## Methods

A cross-sectional descriptive design [24] with a randomly-selected data collection at each level of the cluster sampling (district, sub-district, and healthcare facilities) was performed. Data were collected using questionnaires and each participant was measured one time.

### Setting

The study site was located in a suburban province close to Bangkok, Thailand, with 1,005,760 residents, comprising 52.5% women and 47.5% men, and most of the residents were Buddhist (94.7%) [25]. The catchment areas received medical services from the Health Promoting Hospitals (HPHs), a frontline healthcare service, with free essential treatment cost. These services are provided by the staff of the HPHs in cooperation with the Village Health Volunteers (VHVs), community residents who act as mediators between the staff and the community inhabitants.

### Procedures

#### Sampling method

**Fig. 1:**
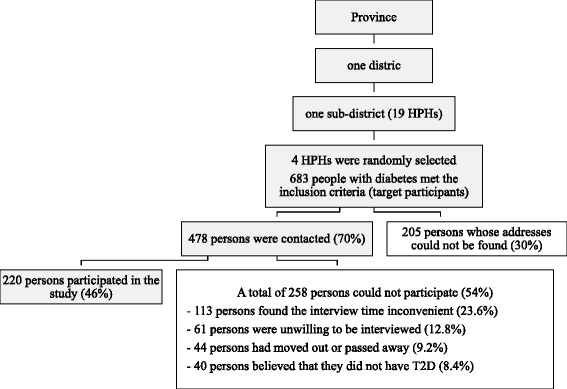
Flow diagram of procedures for selecting the participants

A multistage sampling method [26] was used. One random sample was obtained at each respective level (district, sub-district, and HPH) (Fig. 1). All of the people with T2D that lived in communities within the responsibility of the sampled HPH were invited to participate in this study. If there were not enough participants available in the sampled HPH, another HPH was chosen, and four HPHs took part in this study. The sample size for this study was calculated using the estimated proportion of Thai people with diabetes (7.7%) with the absolute error of 5% and alpha, two-tailed, at 0.05 that the study would have power of 80% to detect the differences of illness perception and self-management among women and men [27]. The minimum sample size, including women and men, was 220 people.

#### Participants

All of the patients shown in the medical records of the HPHs were screened to fit the eligible inclusion criteria for participation: (1) Thai citizens that could converse in the Thai language, (2) individuals diagnosed with T2D by the physician for at least 1 year, and (3) those receiving anti-diabetic agent(s) or insulin until the day of the investigation. The people with T2D admitted to hospitals and those whose address could not be found were excluded from the study. A total of 478 people with T2D were contacted. Of this number, 61 people (12.8%) were not willing to participate in this study, 40 (8.4%) stated that they did not have T2D, 113 (23.6%) were not able to be reached for an interview because they had a daytime occupation (worked every day from 5 am to 8 pm), and 44 (9.2%) had moved out of the area or had died. In total, 220 people with T2D (46% of all contacts) participated in the study (Fig. 1).

#### Data collection

Data collection was run by three different qualified interviewers and supervised by a Thai researcher (first author) from June to August 2015. In order not to disqualify any person that was illiterate, all of the participants were interviewed face-to-face. All of the interviews took place at the participant’s house or a place suggested by the participant. Before the interview began, the study, its purposes, assurance of confidentiality, and how to withdraw from the study were explained. When the participant decided to participate, a consent form was signed. Each interview lasted from 40 to 60 min. If omissions were found afterward, the interviewers visited the participants again to address the incomplete items.

### Measurement tools

The measurement tools comprised a tool measuring the demographic characteristics developed by the researchers and two questionnaires which had earlier showed good psychometric properties: the revised diabetes illness perception (IPQ-R) questionnaire, developed by Moss-Morris et al. [16] (see the illness perception questionnaire website, http://www.uib.no/ipq/), and the 2015 revised diabetes self-management questionnaire (DSMQ-R), developed by Schmitt et al. [28]. Both questionnaires were originally developed in English, and then translated into the Thai language, inspired by the guidelines of the World Health Organization [29]. This main process included forward-backward translation between English and Thai language by two bilingual experts, discussion in a team of researchers to determine inadequate or different concepts of the translation, revision the concepts to be consistent with the original version, and a test of the translated questionnaires with a target population.

The IPQ-R questionnaire used to measure illness perception was divided into three main sections: identity, diabetes perception, and causal sections. The identity section included 14 common symptoms with a yes/no response format. The diabetes perception section consisted of 38 items of cognitive and emotional illness perception when living with T2D, which included seven subscales. Lastly, the causal section included 18 items that measured the participants opinion of what might have been the cause of T2D, focusing on the participants’ own considerations. Both the diabetes perception and causal sections were designed using a five-point Likert scale as follows: 1 (strongly disagree), 2 (disagree), 3 (neither agree nor disagree), 4 (agree), and 5 (strongly agree). High scores for each subscale represented strongly-held or positive beliefs.

The DSMQ-R questionnaire was used to assess the self-care activities over the last 8 weeks of people with T2D. This instrument included 27 items of self-care activities (the first 20 items for non-insulin-treated participants and all 27 items for insulin-treated participants), comprising a sum scale and four sub-scales: glucose management, dietary control, physical activity, and healthcare use. The DSMQ-R was designed based on a four-point Likert scale, ranging from zero to three, with the responses “does not apply to me,” “applies to me to some degree,” “applies to me to a considerable degree,” and “applies to me very much.” The sum for each scale scores was computed and then transformed to a scale, ranging from 0 to 10 [28]. High scores indicated more effective self-management.

#### Psychometric properties of the measurement tools

Both Thai versions of the questionnaires were tested for validity and reliability [30]. The content validity was tested by three nursing experts specializing in diabetes. The process involved a team of experts considering if each item was conformed to the original versions, using 3-point rating scales as follows: 1 (not relevant), 2 (somewhat relevant), and 3 (highly relevant). Answer of each item was transformed into a dichotomous scale where highly relevant was considered as “relevant” and the others referred to “not relevant”. The percentage of each relevant item, given by the experts, were calculated and documented as a content validity index for items (I-CVI) and for scales (S-CVI). [31]

The reliability of the questionnaires was tested for inter-rater, internal consistency, and test-retest. The inter-rater reliability was tested using three people with T2D to develop a consistent understanding among the three interviewers. Each participant was interviewed three times (each time by a different interviewer), and the three sets of questionnaires were checked for inconsistent answers, followed by a consensus discussion between the team of interviewers and the Thai researcher (first author). The internal consistency reliability was measured in a pilot study by interviewing 30 people with T2D. A re-interview was conducted 2 weeks after the initial interview to investigate the test-retest reliability.

### Data analysis

The data were analyzed using SPSS for Windows version 21.0 [32] with a significance level of 0.05.

#### Analysis of the psychometric properties of the instruments

The content validity was established by calculating the content validity index (CVI) [31]. The proportion of each item rated as relevant by three experts, called the I-CVI, was computed, and the S-CVI was obtained by calculating the average of all I-CVIs of each instrument as reported in the Table 1. The percentage of the consistency of each set of questionnaires was calculated in order to determine the inter-rater reliability. The internal consistency reliability was examined using Cronbach’s alpha coefficient [33]. The test-retest reliability was analyzed using Pearson correlation coefficient or the Spearman correlation coefficient, depending on the data distribution [34].

**Table 1:** Validity and reliability test of the measurement tools

Measurement tools	Sum scale/Subscale	Validity and reliability test			
		Content validity index (CVI)	Cronbach’s alpha	Test-retest reliability	
Revised diabetes illness perception questionnaire (IPQ-R)	Identity section	0.98	0.81	r_s_ = 0.697^***^	*p* = 0.000
	Diabetes perception section	0.75	0.76	*r* = 0.502^**^	*p* = 0.005
	Causal section	0.96	0.73	*r* = 0.452^*^	*p* = 0.012
Revised diabetes self-management questionnaire (DSMQ-R)	Sum scale	0.91	0.78	*r* = 0.503^**^	*p* = 0.005

r = Pearson correlation coefficient, r_s_ = Spearman correlation coefficient^, *^ *p* < 0.05 ^**^ *p* < 0.01 ^***^ *p* < 0.001

#### Statistical analysis

The categorical demographic characteristics of the participants were presented according to frequency and percentage, and the median was used for the continuous variables due to a skewed nature [34]. In order to compare the differences between the women and men, a chi-square test was used for the categorical variables, and the Mann-Whitney U test was used for testing the continuous variables [34].

The illness perception was presented according to each section in the IPQ-R scale using percentages, mean, and standard deviation (SD) Additionally, since a non-normal data distribution was found in the identity section and in each subscale of the diabetes perception section, the Mann-Whitney U test was used to compare the distribution of scores between the women and men [34]. In the causal section, each item was transformed and grouped into a dichotomous variable (disagreement—strongly disagree, disagree, neither agree nor disagree; and agreement—agree and strongly agree). The percentage of agreement for each item was calculated to analyze the high respectively low rank of causal agreements.

Diabetes self-management was calculated using the mean score and SD of the sum scale and each subscale. The different scores of all subscales between the women and men were analyzed using the independent samples t test or Mann-Whitney U test, depending on the data distribution [34].

## Results

### Psychometric properties of the instruments

The Thai instruments, which were validated in this study, demonstrated acceptable validity and reliability (Table 1). The content validity of the DSMQ-R scale was high (0.91) and acceptable for all sections of the IPQ-R diabetic version scale (ranging from 0.75 to 0.98). Both instruments met the requirements for internal consistency (Cronbach’s alpha >0.7). For the IPQ-R diabetic version scale, Cronbach’s alpha of subscales were 0.81 for identity section, 0.76 for diabetes perception section, and 0.73 for causal section, and was 0.78 for the sum scale of the DSMQ-R instrument. The percentages of the consistency concerning inter-rater reliability ranged from 84.6 to 94.2%, which reflected consistent understanding in the team of interviewers. The results of the test-retest reliability showed a moderate association at the different significance level for both instruments, with the correlation coefficients ranking between 0.452 and 0.697, and the *p*-value in a range between less than 0.001 and less than 0.05. Results showing a moderate stability of the instruments obtained on two separate occasions.

### Characteristics of the participants

The demographic characteristics are shown in Table 2. Of the total 220 participants, there were 150 women (68.2%) and 70 men (31.8%), and all were Buddhists. Most of the participants were married and had completed their education at the primary school level. More than half of the participants were unemployed. Men showed a higher percentage of marriage status (χ^2^ = 5.344, *p* = 0.021, df = 1), had a higher educational level (χ^2^ = 25.271, *p* < 0.000, df = 3), were more often employed (χ^2^ = 5.605, *p* = 0.018, df = 1), and were significantly older than women (Z = −2.370, *p* = 0.018). Regarding the illness-related characteristics, most of the participants took oral anti-diabetic agents, used a universal coverage program as their preferential treatment, and received health services at the HPH and other public hospitals. There were no significant differences between men and women according to these illness-related characteristics, except for a higher incidence of diabetic complications among women (χ^2^ = 4.314, *p* = 0.038, df = 1).

**Table 2:** Demographic characteristics of the participants

Demographic variables	Total (*n* = 220) *n* (%)	Women (*n* = 150) *n* (%)	Men (*n* = 70) *n* (%)	Statistical test	*p*-value
1. Socio-demographic characteristics					
Educational level					
Unschooled	27 (12.3)	24 (16.0)	3 (4.3)	χ^2^ = 25.271^***^	0.000
Primary school (Pratom 1 to 6)	146 (66.4)	107 (71.3)	39 (55.7)		
Secondary school (Mathayom 1 to 6)	30 (13.6)	14 (9.3)	16 (22.9)		
Higher than secondary school	17 (7.7)	5 (3.3)	12 (17.1)		
Marital status					
Married	146 (66.4)	92 (61.3)	54 (77.1)	χ^2^ = 5.344^*^	0.021
Not married	74 (33.6)	58 (38.7)	16 (22.9)		
Religion					
Buddhism	220 (100.0)	150 (100.0)	70 (100.0)	–	–
Occupation					
Employed	94 (42.7)	56 (37.3)	38 (54.3)	χ^2^ = 5.605^*^	0.018
Unemployed	126 (57.3)	94 (62.7)	32 (45.7)		
2. Illness-Related information					
Ordinary health service use					
Health Promoting Hospital	109 (49.5)	70 (46.7)	39 (55.7)	χ^2^ = 1.742	0.419
Other public hospitals	94 (42.7)	67 (44.7)	27 (38.6)		
Private Hospital	17 (7.7)	13 (8.7)	4 (5.7)		
Preferential treatment^a^					
Universal coverage	173 (78.6)	123 (82.0)	50 (71.4)	χ^2^ = 3.213	0.201
Other preferential treatment	37 (16.8)	21 (14.0)	16 (22.8)		
Self-payment	10 (4.5)	6 (4.0)	4 (5.7)		
Current treatment					
Oral anti-diabetic agent(s)	183 (83.2)	124 (82.7)	59 (84.3)	χ^2^ = 0.089	0.765
Oral pills in combination with other treatments	37 (16.8)	26 (17.3)	11 (15.7)		
Experience of diabetes complications					
No	53 (24.1)	30 (20.0)	23 (32.9)	χ^2^ = 4.314^*^	0.038
Yes	167 (75.9)	120 (80.0)	47 (67.1)		
Median (interquartile)					
Age (year)	64 (55-70)	62.5 (54-69.25)	67 (59.25-73)	Z = −2.370^*^	0.018
Duration of illness (year)	8 (4-14.5)	8 (4-14)	8.5 (3.75-15)	Z = −0.515	0.607
Level of Fasting Plasma Glucose (mg/dl)	144 (121.25-184)	147 (120-190)	141 (126-177)	Z = −0.671	0.502

^a^ receive the treatment paid by the civil servants’ medical benefits, social security, or universal coverage scheme

^*^ *p* < 0.05, ^**^ *p* < 0.01, ^***^ *p* < 0.001, χ^2^ = Chi-square, Z = Mann-Whitney U test

### Illness perception

The 50th percentile (median) of symptoms experienced after being diagnosed with T2D was five (median (Mdn) 5, interquartile range (IQR) 3-7), six for women (Mdn 6, IQR 4-8) and three for men (Mdn 3, IQR 2-6). The most common symptoms that women experienced were dizziness, fatigue, pain, weight loss, sleep difficulties, and loss of strength, whereas men most often experienced fatigue, dizziness, and weight loss. Of these symptoms, both sexes believed that two symptoms were related to their diabetes (women: Mdn 2, IQR 1-4; men: Mdn 2, IQR 1-4), with no significant differences concerning the number of symptoms related to diabetes.

As seen in Table 3, women perceived to a higher degree negative consequences of T2D (Z = −2.204, *p* = 0.028) and a more fluctuating nature of their disease (Z = −3.441, *p* = 0.001) than men. Men felt more confident in the treatment given by the health professionals than women (Z = −2.031, *p* = 0.042).

**Table 3:** Tests for the different mean scores of illness perception between women and men

Subscales	Range	Total (*n* = 220) Mean (SD)	Women (*n* = 150) Mean (SD)	Men (*n* = 70) Mean (SD)	Mann-Whitney U test	*p*-value
Acute or chronic conditions^a^	5-30	24.53 (3.83)	24.75 (3.56)	24.07 (4.34)	Z = −0.516	0.606
Consequences	5-30	13.45 (3.88)	13.85 (3.96)	12.60 (3.60)	Z = −2.204*	0.028
Personal control	5-30	24.94 (2.71)	24.83 (2.74)	25.17 (2.67)	Z = −0.984	0.325
Treatment control	5-25	19.16 (2.52)	18.95 (2.55)	19.61 (2.41)	Z = −2.031*	0.042
Illness coherence	5-25	17.88 (3.43)	17.65 (3.60)	18.36 (3.00)	Z = −1.134	0.257
Fluctuating symptoms^b^	5-20	9.83 (2.82)	10.29 (2.73)	8.84 (2.76)	Z = −3.441**	0.001
Emotional representation	5-30	11.02 (5.16)	11.33 (5.38)	10.37 (4.63)	Z = −1.071	0.284

^a^Subscale name “timeline” was changed to “acute or chronic condition”

^b^Subscale name “timeline cyclical” was changed to “fluctuating symptoms”

^*^ *p* < 0.05, ^**^ *p* < 0.01

Women and men presented a high percentage of agreement for possible causes of T2D. These causes were diet or eating habits (women: 85.3%, men: 92.9%), personal behaviors (women: 68.7%, men: 71.4%), and poor medical care in the past (women: 65.3%, men: 72.9%).

### Self-management

There was no significant overall difference found in the self-care activities between women and men, although women demonstrated higher mean scores of glucose management and of healthcare use than men and men showed higher mean scores of dietary control and of physical activity than women (Table 4).

**Table 4:** Tests for the different mean scores of self-management between women and men

Subscales	Total (*n* = 220)	Women (*n* = 150)	Men (*n* = 70)	Statistical tests	*p*-value
	Mean (SD)	Mean (SD)	Mean (SD)		
Sum scale	7.11 (1.24)	7.07 (1.16)	7.20 (1.41)	*t* = −0.680	0.498
Subscale					
- glucose management	6.80 (1.29)	6.83 (1.19)	6.73 (1.48)	Z = −0.055	0.956
- dietary control	7.34 (1.86)	7.26 (1.84)	7.51 (1.91)	Z = −0.978	0.328
- physical activity	7.13 (2.18)	6.97 (2.14)	7.46 (2.25)	Z = −1.718	0.086
- healthcare use	7.97 (1.34)	7.98 (1.21)	7.94 (1.58)	Z = −0.784	0.433

t = Independent samples t test, Z = Mann-Whitney U test

## Discussion

Our findings showed that there were some differences in the illness perception between the Thai women and men with T2D, while they had similar experiences regarding self-management. Furthermore, the translated instruments used in this study showed acceptable content validity and internal consistency reliability, and moderate test-retest reliability.

The women in this study, in accordance with earlier studies, perceived to a higher degree negative consequences of the disease [12, 17, 18] as well as fluctuating symptoms than men [35]. They also showed a higher blood sugar level than the standard recommendations of glycemic control for adults [36], implying poor glycemic control, which is known to lead to several symptoms connected with diabetes complications [37]. Furthermore, women in earlier studies were shown to be worried about their unpredictable future with diabetes [38] and expected everything to be under their control [9, 39], which may lead them to express negative perceptions when they detect something that does not align with their expectations.

On the other hand, men in this study showed higher confidence in the treatment effectiveness than women. This might be related to their higher educational level, corresponding to the findings in earlier research where it was seen that education may enhance the individual’s sense of control, knowledge, and life skills [40, 41]. Furthermore, men in our study assessed their experiences of diabetes complications to a lower degree, which might give them a greater opportunity to develop confidence in the treatment, as seen in earlier studies [11, 12].

Our findings, that both women and men showed a low score on the “emotional representation” subscale for illness perception, is interesting since it might point to a less emotional response in a Thai diabetes population in comparison with Western populations [6, 8–10]. This might reflect that there is a different view of diabetes among people from diverse social contexts. While most Western people are shown to hold a strong belief in the biomedical model and to focus on self-responsibility [42], research conducted with Eastern people has found that they place a high value on a holistic view of health and illness, connecting with family closeness and religious beliefs [43] and social norms and values [44]. Corresponding to previous studies of Thai women with diabetes, the close attentiveness, encouragement, and understanding among the members in Eastern families [45] and their Buddhist beliefs [46] may have empowered our participants to improve their mental strength and have enabled them to accept their fate and to remain calm. This may also have increased their ability to cope with and manage their daily routines to suit their lives, in accordance with earlier Eastern research [47, 48].

Another finding from this study, contrary to earlier Western research [15, 49], was the similarity in dietary control of women and men. A difference that might be explained by the influence earlier found from Buddhist teaching, the idea of using moderation and balance to accomplish goal existing in reality [50], and the strategy of “eating in moderation” in order to maintain acceptable levels of plasma glucose for most patients with T2D [47, 48]. Holding this view may have persuaded our participants with T2D to select proper food, reduce their intake of sweets, and to control their craving for harmful foods. Another explanation might be that patients with T2D in Thailand experience an inferior status to the healthcare professionals in the Thai healthcare system and tend to follow their advice [51]. Additionally, the personality of the Thai people, who choose avoidance or compromise rather than confrontation [52], may also have impelled the participants regardless of gender to comply with medical suggestions and to try to be consistent regarding the recommendations given to them.

The finding that both women and men demonstrated a low score on the glucose management subscale, which focused on self-monitoring of blood glucose levels (SMBG) and taking diabetes medications, was also interesting. Although SMBG was found to be a common self-care practice for managing diabetes among Western people [15], this procedure is not widely used among Thai people with T2D. In Thailand, healthcare professionals tend to take responsibility to monitor monthly plasma glucose, and SMBG seems to be inaccessible for Thai patients, at least our participants. Additionally, no formal patient education was provided at the HPHs; hence, the participants had less opportunity to learn about glucometers.

Being aware that the diabetes perceptions and the influences of the social context in the Thai population are gender based, might influence healthcare professionals to design clear, concise, and specific patient education programs. For an Eastern population with T2D, the concept of holistic care and belief in Buddhist teaching can be used to promote more effective self-management, particularly dietary control. Including assessments of diabetes perceptions and self-care activities, as well as considering the preferences, needs, and beliefs of patients with T2D, might increase the compliance of supportive programs for individuals of both sexes in this population.

### Strengths and limitations

Our findings were interpreted to have both strengths and limitations. The homogeneous socio-demographic characteristics of the participants, who were recruited from the same catchment areas of the HPH, were considered, and randomly selecting the HPHs was used to reduce bias and to provide an equal chance for each HPH to be chosen as a study sample [27]. This may allow our findings to be generalized to people with diabetes living in the suburban areas around Bangkok, Thailand. Additionally, our estimation of participating numbers of women and men calculated based on the likelihood ratio of developing T2D in Thai women and Thai men.

(2 : 1) [5] may also increase the possibility to generalize the findings to other populations.

The structured interview method provided an opportunity to obtain answers from all literate and illiterate participants. Furthermore, the researchers tried to reduce the errors in the data collected by selecting experienced interviewers, training them before the interview, and coaching them with the first author once a week. However, we lost one-fifth of the potential participants that were occupied with a daytime job, which would provide additional information. In addition, the relatively high degree of dropout participants (30%), with inaccurate addresses, might be a drawback to influence our results. However, it is a factor out of our control since finding accurate address in the Thai system is difficult and complex. The Thai version of the instruments used in this study showed acceptable validity and reliability, which showed that they were the proper tools for collecting the data, but these questionnaires still had some restrictions. For example, the glucose management subscale in the DSMQ-R questionnaire, which has three items focusing on SMBG activities, may not be suitable for assessing information from participants that do not use a glucometer. Furthermore, only 16 insulin-treated participants took part in this study, and these results should not be generalized to the insulin-treated population.

## Conclusion

This study of women and men living with T2D in a suburban area of Bangkok, Thailand, showing that there are many similarities but also some differences between women and men in illness perception, might provide new knowledge in this area of research. Furthermore, the lack of differences between men and women with T2D regarding self-care activities in a Thai population is different from Western studies. The findings that women perceived more negative consequences of diabetes and more fluctuating symptoms of their disease, while men perceived more confidence in treatment effectiveness, are of interest and consistent with earlier research.

## Abbreviations

SMBG: Self-monitoring of blood glucose levels
T2D: Type 2 diabetes
